# Universal Parafilm^®^-Mediated Thermal Bonding for Gold Leaf Electrode Patterning and Microfluidic Assembly on Diverse Substrates

**DOI:** 10.3390/mi17101136

**Published:** 2026-09-29

**Authors:** Zhiyuan Yang, Yafei Lou, Siyu Chen, Yongye Chen, Linan Sun, Junfei Tian, Rong Cao

**Affiliations:** 1 NHC Key Laboratory of Tropical Disease Control, School of Life Sciences and Medical Technology, Hainan Medical University, Haikou 571199, China; 2 State Key Laboratory of Pulp and Paper Engineering, School of Light Industry and Engineering, South China University of Technology, Guangzhou 510640, China

**Keywords:** paper-based sensor, microfluidic device, electrochemical detection, gold leaf electrode, Parafilm^®^ sheet

## Abstract

Gold leaf electrodes offer a low-cost, substrate-versatile strategy for electrochemical sensing and microfluidic systems. Herein, we demonstrate a unified Parafilm^®^-mediated platform in which the Parafilm^®^ layer serves as both the adhesive for gold-leaf electrode patterning and the bonding/channel-defining layer for microfluidic assembly via heat-activated bonding. Gold leaf electrodes were patterned on diverse substrates by laser ablation, achieving a minimum achieved linewidth of 53 ± 3 μm on polyethylene terephthalate (PET) sheets. Electrochemical characterization showed stable CV responses after 40 manual bending cycles and good batch reproducibility. Leveraging Parafilm’s hydrophobicity and thermoplasticity, we integrated electrodes into hierarchical microfluidic architectures on paper and PET. Paper-based hybrid devices with asymmetric self-driven channels enabled dual-zone measurements. Structured PET microchannels yielded microfluidic devices with sequential flow control and selective loading. In a proof-of-concept H_2_O_2_ assay, on-chip Prussian blue electrodeposition followed by amperometry gave a detection limit of 0.44 mg/L and ~85% recovery in spiked pond water. This Parafilm^®^-mediated strategy provides a low-cost route for cross-substrate microfluidic–electrochemical integration, unifying electrode fabrication and device assembly on a single thermoplastic bonding platform.

## 1. Introduction

Gold electrodes serve as a cornerstone material in electrochemical sensing owing to their exceptional electrical conductivity, superior chemical stability, and remarkable biocompatibility [1]. Their unique surface properties, particularly the facile functionalization enabled by gold–thiol chemistry, provide a versatile platform for immobilizing biorecognition elements such as antibodies and oligonucleotides [2,3]. This has led to widespread applications in biomedical diagnostics, food safety, and environmental monitoring. Currently, advances in ultrathin, patterned gold electrodes are driving innovations in flexible electronics and microfluidics, with emerging applications in wearable devices and miniaturized analytical systems [4,5,6].

Conventional fabrication of gold electrodes relies on thin-film deposition techniques, such as physical or chemical vapor deposition, and often additionally requires lithographic patterning and etching [1,7,8]. Although these methods yield high-performance electrodes, they face challenges such as complex processing, high equipment costs, and long fabrication cycles. Furthermore, such techniques often require cleanroom facilities and involve hazardous chemicals, posing significant environmental and safety risks [9]. To overcome these limitations, recent studies have shifted toward cost-effective alternatives such as inkjet-printed or screen-printed gold electrodes, which enable rapid fabrication, reduce material waste, and eliminate reliance on sophisticated infrastructure [10,11].

Meanwhile, alongside continued efforts to optimize gold ink formulations for printed electrodes, researchers are redirecting attention to commercially available gold leaf—a simple yet versatile material—as a promising alternative for facile electrode fabrication [12,13,14,15]. These gold leaf electrodes have already demonstrated notable advantages in cost efficiency, streamlined prototyping workflows, and superior compatibility with flexible electronics. Current fabrication techniques employ thermal lamination [12], electrostatic interaction [13], or adhesives (glues and tapes) [14,15] to adhere gold leaf to diverse substrates, often coupled with blade plotting or laser ablation for electrode patterning. These electrodes now serve diverse functions, ranging from viral sensing on transparency sheets to wearable energy storage on polyester textiles and photovoltaic interfaces on fluorine-doped SnO_2_ glass, which highlights their broad, cross-disciplinary potential [13,16,17]. These expanding applications call for a universal strategy to adhere gold leaf across substrates ranging from rigid to flexible, which would ensure cross-material compatibility and streamline final device assembly.

**Figure 1:**
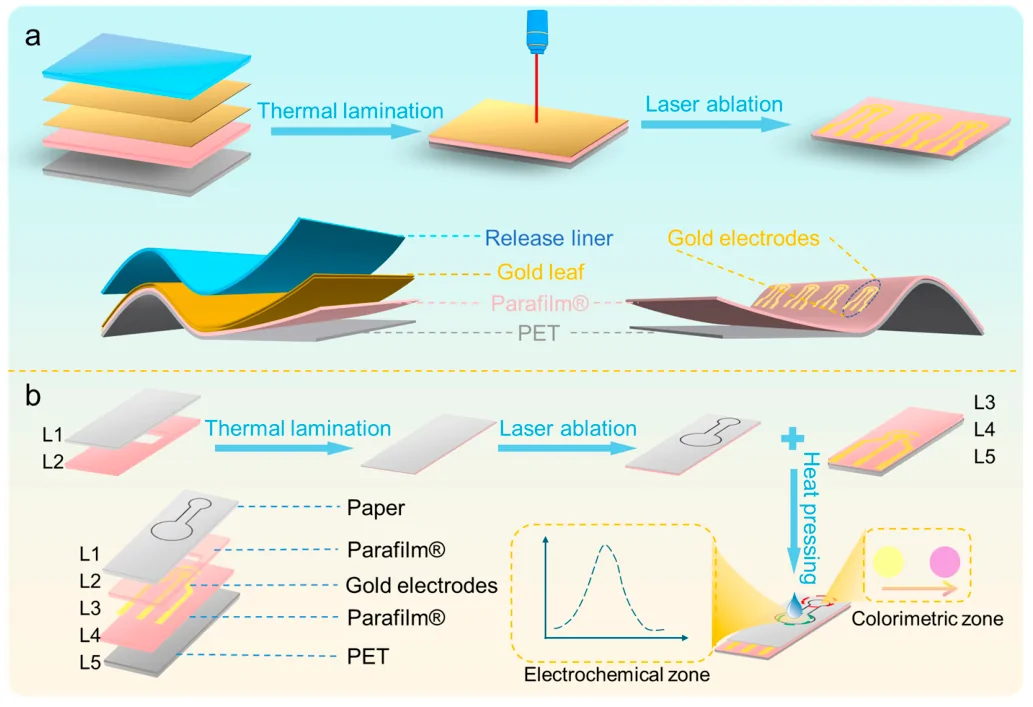
Schematic illustration of (**a**) the fabrication process for gold leaf electrodes and (**b**) the paper-based hybrid microfluidic device integrating gold leaf electrodes for dual-mode colorimetric and electrochemical sensing, showing the layered architecture (L1–L5).

Parafilm^®^ is valued for its cost-effectiveness, chemical resistance, high pliability, and water repellency. Notably, its thermoplastic properties enable robust adhesion across diverse materials, ranging from rigid glass to flexible paper, making it ideal for microfluidic device fabrication [18,19,20,21]. A pertinent question arises: can this heat-activated adhesion be extended to gold leaf, turning Parafilm^®^ into a universal electrode adhesive? If feasible, Parafilm^®^ could simultaneously define microfluidic channels while thermally bonding gold leaf electrodes to substrates. This fabrication strategy enables the rapid prototyping of microfluidics integrated with electrochemical electrodes through a simple, streamlined process, and can be readily adapted to diverse microfluidic–electrochemical platforms. The central advance of this work is the use of Parafilm^®^ as both a thermoplastic electrode adhesive and a microfluidic bonding/channel-defining layer. As illustrated in Figure 1a, Parafilm^®^ was utilized as an adhesion layer to bond gold leaf onto polyethylene terephthalate (PET) substrates during electrode fabrication. Patterned gold leaf electrodes were then created by laser ablation. Benefiting from the compatible Parafilm^®^ interfaces, these electrodes can be thermally laminated with diverse Parafilm^®^-defined microfluidic constructs (e.g., on PET or paper) via a simple heat-pressing process. As a representative example, Figure 1b demonstrates the integration of Parafilm^®^-defined paper microfluidics with the patterned gold leaf electrodes, yielding a hybrid microfluidic device capable of simultaneous colorimetric and electrochemical sensing. Our strategy establishes a versatile platform that spans substrates from flexible to rigid (PET, fabric, paper, ceramic, glass) and accommodates interchangeable metal foils, enabling integration of electrodes into diverse microfluidic systems.

## 2. Materials and Methods

### 2.1. Reagents and Instruments

Uric acid (UA), potassium ferricyanide (K_3_Fe(CN)_6_), potassium ferrocyanide (K_4_Fe(CN)_6_), potassium chloride (KCl), L-ascorbic acid, glucose, phosphate-buffered saline (PBS), and hydrogen peroxide (H_2_O_2_) standard solution were purchased from Macklin Biochemical Co., Ltd. (Shanghai, China). Bromophenol red sodium salt and dopamine hydrochloride were purchased from Sangon Biotech Co., Ltd. (Shanghai, China). Anhydrous iron(III) chloride (FeCl_3_) was obtained from Aladdin Reagent Co., Ltd. (Shanghai, China). Hydrochloric acid (HCl) and sulfuric acid (H_2_SO_4_) were obtained from Guangzhou Chemical Reagent Factory (Guangzhou, China). All reagents were used as received, and ultrapure water (18.2 MΩ·cm) was used throughout the experiments. PET film, 300 μm thick, was obtained from Tianfu Plastic Products Co., Ltd. (Dongguan, China). Gold leaf, silver foil, and copper foil were supplied by Nanjing Yongbo Metal Materials Co., Ltd. (Nanjing, China). Silver/silver chloride (Ag/AgCl) paste was obtained from Shenzhen Yi Lai Technology Co., Ltd. (Shenzhen, China). Parafilm^®^ M sealing film (PM-996) with a thickness of 127 µm was used in the experiments. A thermal laminator (GQ190, Deli, Ningbo, China) and a heat press (HPM10, Loklik, Changsha, China) were employed for thermal bonding processes. A cutting plotter (S500, Purcell, Shenzhen, China) was used to engrave patterned designs on Parafilm^®^ and PET films. A portable laser engraver (F1, xTool, Shenzhen, China) fitted with 10 W diode (455 ± 15 nm) and 2 W infrared (1064 ± 5 nm) sources was used to pattern gold leaf, silver foil, and copper foil. The surface morphology of the gold leaf electrodes was analyzed by scanning electron microscopy (SEM, SU8600, Hitachi, Tokyo, Japan). An electrochemical workstation (CHI660E, Chenhua, Shanghai, China) was employed to conduct cyclic voltammetry (CV), differential pulse voltammetry (DPV), and electrochemical impedance spectroscopy (EIS) analyses.

### 2.2. Fabrication of Gold Leaf Electrodes

The gold leaf electrodes were fabricated through a layer-by-layer assembly and laser ablation process (Figure 1a). Initially, a multilayer stack was sequentially constructed as follows: a PET film as the substrate, a Parafilm^®^ layer, two overlapping gold leaf layers, and a release liner. Although a single gold leaf layer is electrochemically sufficient, we employed two overlapping layers to improve surface uniformity by covering cracks and holes that readily form when a single layer is transferred [2]. Control experiments confirmed that single- and double-layer electrodes were electrochemically comparable: cyclic voltammograms in 10 mM K_4_[Fe(CN)_6_]/1 M KCl showed nearly identical peak currents and peak shapes, with negligible background response in PBS (Figure S1). Dual gold-leaf layers were employed as the standard configuration unless otherwise specified. The multilayer stack was then laminated using the thermal laminator at the 125-mic setting and cooled to room temperature. The short contact time could make the Parafilm^®^ interlayer tacky enough to adhere via interfacial wetting rather than bulk flow. After removing the release liner, selective ablation of the gold leaf was carried out with an infrared laser (1064 ± 5 nm, 2 W) at 300 mm/s, 32% power, 2 passes, 300 Dots Per Inch, bi-directional fill engraving. The dimensional accuracy of the laser-ablated gold leaf patterns was evaluated with an optical microscope (BX51, Olympus, Tokyo, Japan). Their electrical resistance was measured using a digital multimeter (DEM P0, Delixi, Yueqing, China). All versatility tests, including those evaluating material diversity (gold leaf, silver, and copper foils) and substrate generality (fabric, paper, ceramic, glass), were conducted with single-layer metal foils. A blue-light laser source (455 ± 15 nm, 10 W) was exclusively adopted to achieve ablation on silver and copper foils. Ceramic and glass were incompatible with the thermal laminator, so the heat press was used to bond the gold leaf onto these rigid substrates through Parafilm^®^.

### 2.3. Electrochemical Evaluation of Gold Leaf Electrodes

Typically, CV of the gold leaf electrodes was performed in 0.5 M H_2_SO_4_ over a potential range from −0.1 V to 1.6 V at 0.1 V/s. In a solution containing 10 mM K_4_[Fe(CN)_6_] and 1 M KCl, the gold leaf electrodes were subjected to CV cycles between −0.2 V and 0.6 V, and CV curves were recorded at varying scan rates from 0.05 V/s to 0.5 V/s in increments of 0.05 V/s. EIS was conducted in 0.1 M KCl containing 10 mM K_3_[Fe(CN)_6_]/K_4_[Fe(CN)_6_] as the redox couple. Measurements were performed over a frequency range of 0.01 Hz to 100 kHz with an amplitude of 5 mV. DPV was conducted on serial dilutions of UA (10 μM–1 mM) within a potential range of 0.1 V to 0.6 V, using a pulse amplitude of 0.05 V and a pulse width of 0.05 s.

### 2.4. Fabrication and Characterization of Microfluidic–Electrochemical Hybrids

The paper-based hybrid device features a five-layer architecture, with schematics of each layer provided in Figure S2. Using a Parafilm^®^-heating-laser-cutting protocol as previously described [19], Whatman qualitative filter paper (Grade 1, Cytiva, Hangzhou, China) was thermally bonded with Parafilm^®^, followed by laser ablation of the paper layer to create asymmetric, dumbbell-shaped microfluidic channels. Concurrently, a three-electrode system was fabricated by laser-patterning gold leaf thermally laminated to PET. The microfluidic layers (L1, L2) and electrode layers (L3–L5) were then aligned, with Parafilm^®^ interlayers acting as thermally activated adhesives, and fused by the heat press machine to complete the integrated device. In-situ DPV measurements and pH sensing (3 mM bromophenol red as the pH indicator) were performed on the device after adding 1 mM UA in 0.01 M PBS.

The PET-based microfluidic–electrode hybrid device comprises a nine-layer laminated structure, with schematics of each layer shown in Figure S3. The bottom three independent three-electrode systems were created by laser-patterning gold leaf thermally bonded to PET via a Parafilm^®^ interlayer. The reference electrodes were then coated with Ag/AgCl paste and oven-dried, converting them to Ag/AgCl. The alternating Parafilm^®^ and PET films containing plotter-cut microchannel networks were stacked sequentially and capped with a PET cover bearing a reagent inlet, three sample inlets, and three waste outlets. The heat press was used to fuse the assembly into a transparent microfluidic–electrochemical system where Parafilm^®^ interlayers simultaneously define sealed channels and supply adhesive integrity. To modify the gold leaf working electrode, a solution containing 2 mM FeCl_3_, 2 mM K_3_[Fe(CN)_6_], 0.2 M KCl, and 5 mM HCl was introduced from the reagent inlet, and Prussian blue (PB) was electrodeposited at +0.4 V for 100 s. After that, a supporting electrolyte of 0.1 M KCl and 0.1 M HCl was introduced, and the electrode was stabilized by CV (−0.2 to 0.4 V, 0.05 V/s, 20 cycles). A series of H_2_O_2_ standard solutions in 0.1 M KCl and 0.01 M HCl was introduced from the sample inlet, and amperometric detection was performed at a constant potential of −0.1 V for 50 s. Filtered pond water aliquots were spiked with H_2_O_2_ to 2 mM, 1 mM, and 500 μM, then diluted 10-fold with the acidic supporting electrolyte (0.1 M KCl + 0.01 M HCl), and introduced from the sample inlet for amperometric detection. This acidification/dilution step was used to stabilize the PB film. The reported recovery refers to acidified, diluted pond water rather than direct neutral-water analysis.

## 3. Results and Discussion

### 3.1. Fabrication and Characterization of Laser-Patterned Gold Leaf

**Figure 2:**
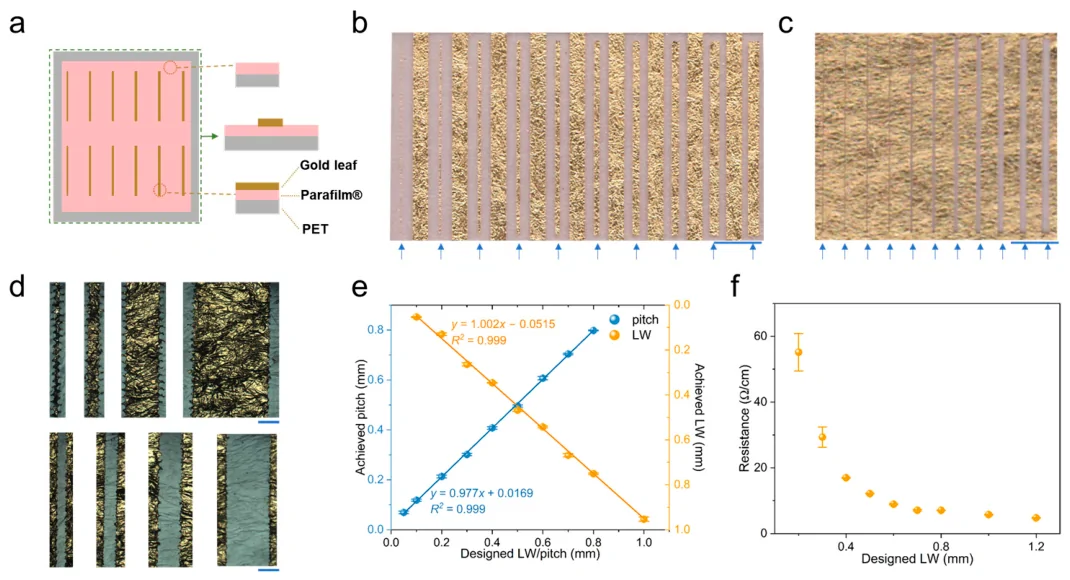
(**a**) Top-view and side-view schematic illustration of the patterned gold leaf. (**b**) Laser-ablated gold leaf line patterns with designed widths (left to right: 0.1–0.8 mm in 0.1 mm increments, 1.0 mm, and 1.2 mm). Scale bar: 5 mm. (**c**) Laser-ablated patterns with designed pitches (left to right: 0.05 mm, 0.06 mm, 0.08 mm, and 0.1–0.8 mm in 0.1 mm increments). Scale bar: 5 mm. (**d**) Optical images of laser-ablated patterns with designed dimensions. Top row (left to right): line widths of 0.1, 0.2, 0.4, and 0.8 mm; bottom row (left to right): pitches of 0.05, 0.1, 0.2, and 0.4 mm. Scale bar: 0.2 mm. (**e**) Plots showing correlation between designed and achieved dimensions of LW and pitch (*n* = 3). (**f**) Plot showing the change in linear resistance (Ω/cm) as a function of LW (*n* = 3).

To validate Parafilm^®^’s feasibility as a versatile adhesive interlayer, we began our investigation with PET, a widely used flexible substrate in wearables and microfluidics. The fabrication process started with bonding gold leaf to the PET surface by thermally laminating a Parafilm^®^ interlayer, followed by precise patterning with laser ablation. As shown in Figure 2a, the unablated regions retained the original PET/Parafilm^®^/gold layered architecture. Conversely, the laser-ablated regions showed complete gold leaf removal, exposing the underlying Parafilm^®^ surface without compromising PET substrate integrity. To systematically characterize the dimensional accuracy and process precision of laser patterning, we conducted a quantitative evaluation of line width (LW) and line pitch control capabilities. Herein, “designed dimension” refers to the dimension specified in the laser ablation file; “achieved dimension” refers to the dimension measured after laser ablation. Figure 2b and c demonstrate the laser-ablated linear arrays with progressively scaled dimensions, in which the designed LWs span 100–1200 μm and the designed pitches range from 50 to 800 μm. The process reliably generated the patterns across these ranges. Optical microscopy revealed a systematic reduction in the achieved LW compared to the designed LW (Figure 2d and Table S1). Quantitatively, the achieved LWs were consistently 32–70 μm narrower than the designed values across the entire test range (Table S1). Despite this deviation, a linear correspondence existed between achieved and designed LWs across the entire test range (Figure 2e), suggesting that a linear correction factor could be applied to offset the discrepancy. In contrast, the achieved pitch closely matched the designed pitch (Figure 2d and Table S2), with a strong linear correlation observed in Figure 2e. For designed pitches ≥300 μm, the absolute deviation was below 10 μm (relative deviation <3%). The minimum achieved feature sizes obtained were 53 ± 3 µm for LW (designed LW: 100 µm) and 69 ± 6 µm for pitch (designed pitch: 50 µm).

**Figure 3:**
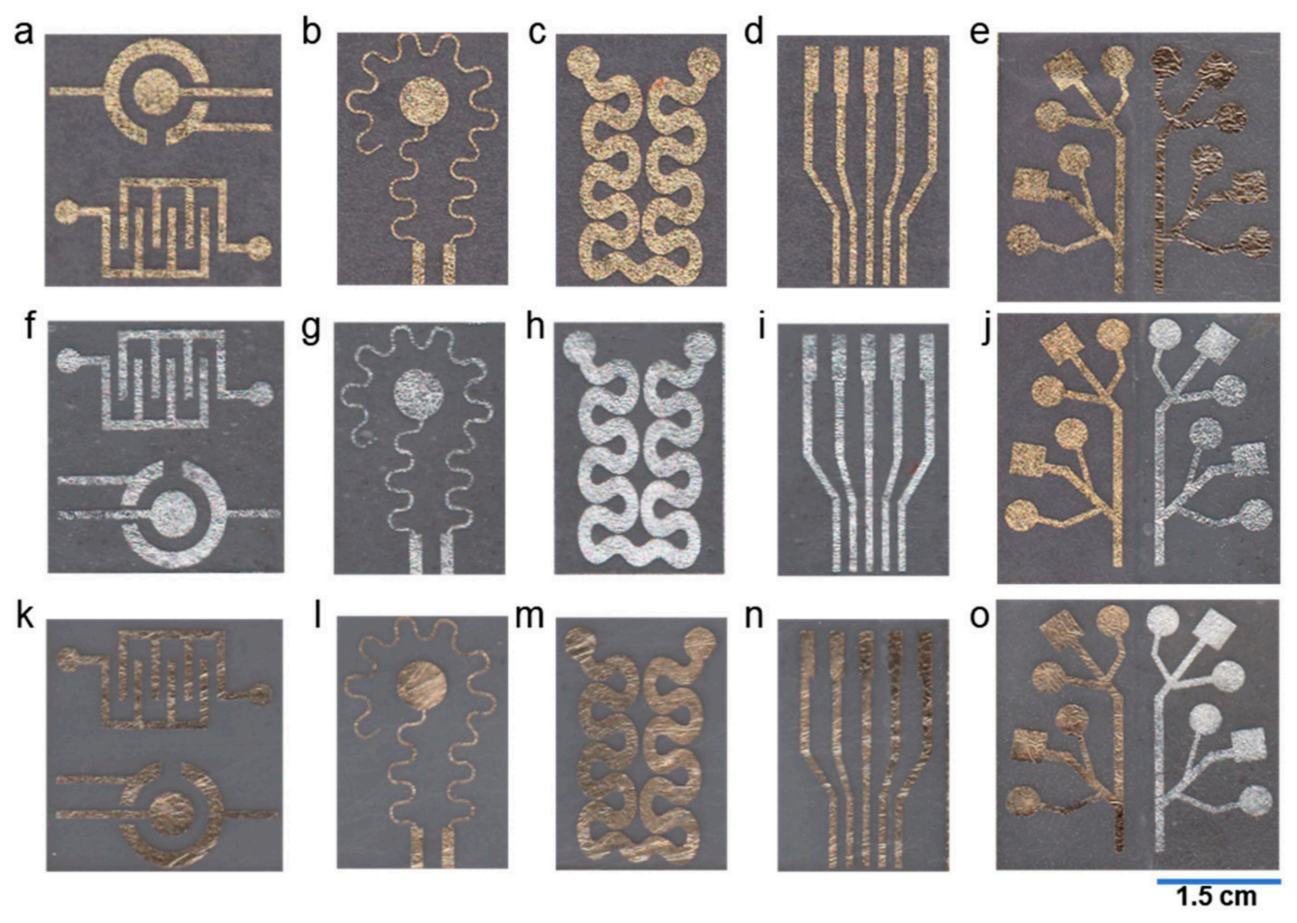
(**a**–**d**) Laser-ablated gold leaf electrode patterns of various geometries on PET. (**e**) Laser-ablated dendritic pattern with gold leaf and copper foil. (**f**–**i**) Laser-ablated silver foil electrode patterns of various geometries on PET. (**j**) Laser-ablated dendritic pattern of gold leaf and silver foil. (**k**–**n**) Laser-ablated copper foil electrode patterns of various geometries on PET. (**o**) Laser-ablated dendritic pattern of copper foil and silver foil.

Further electrical characterization indicated that the smallest designed LW required for reliable electrical conductivity was 200 μm (Figure 2f). The resistance exhibited a decreasing trend as the LW increased. When the LW exceeded 600 μm, the resistance stabilized below 8 Ω/cm, with no significant variation observed across wider line profiles. This observed trend aligns with previous findings by Patel et al. and Sheng et al. [5,15], demonstrating consistency in fundamental conductive behaviors despite differences in the fabrication techniques of patterned gold leaf. In addition, to validate the versatility of the proposed fabrication strategy, we extended its application to diverse metal foils. Using the Parafilm^®^-mediated bonding and laser ablation process, we successfully fabricated gold, silver, and copper electrodes with precisely defined geometries (e.g., interdigital and serpentine electrodes) on PET substrates (Figure 3). Remarkably, dendritic patterns with spatially segregated bimetallic domains (i.e., Au/Cu, Au/Ag, and Cu/Ag) were fabricated by laterally pairing different metal foils on the Parafilm^®^–PET stack, followed by thermal lamination and selective laser ablation (Figure 3e,j,o). These results establish the proposed fabrication approach’s potential for constructing multimaterial integrated devices requiring heterogeneous functional architectures.

### 3.2. Electrochemical Characterization of Gold Leaf Electrodes

**Figure 4:**
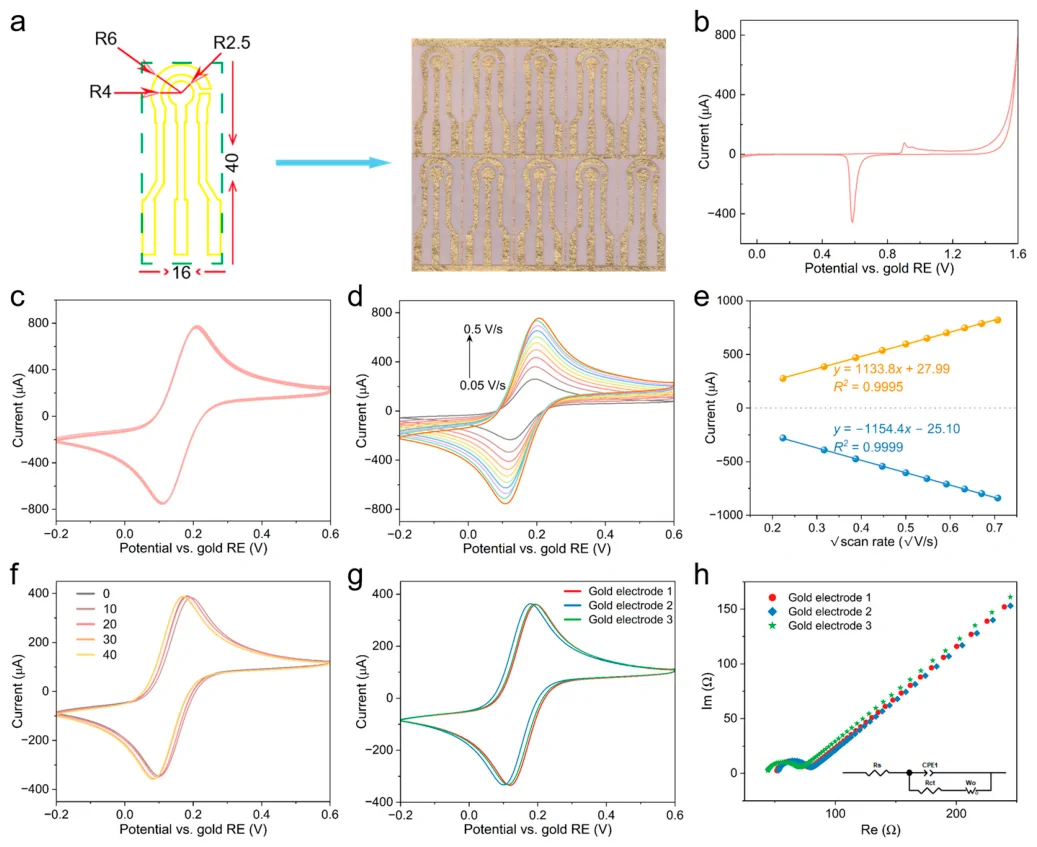
(**a**) Schematic illustration of the gold leaf electrodes (dimensions in mm; R denotes radius) and optical images of the electrodes. (**b**) Cyclic voltammogram of the gold leaf electrodes in 0.5 M H_2_SO_4_ at 0.1 V/s. (**c**) Overlaid cyclic voltammograms (40 consecutive scans at 0.5 V/s) of the gold leaf electrodes in 10 mM K_4_[Fe(CN)_6_]/1 M KCl. (**d**) Cyclic voltammograms recorded at scan rates ranging from 0.05 V/s to 0.5 V/s. (**e**) Linear dependence of anodic and cathodic peak currents on the square root of scan rate. (**f**) Cyclic voltammograms of the gold leaf electrodes at 0.1 V/s before bending (0 cycles) and after 10, 20, 30, and 40 manual bending cycles. The bending procedure involved bringing the electrode end into contact with the wire end, followed by release. (**g**) Cyclic voltammograms of three independently prepared gold leaf electrodes at 0.1 V/s. (**h**) Nyquist plots of three independently prepared gold leaf electrodes in 0.1 M KCl with 10 mM K_3_[Fe(CN)_6_]/K_4_[Fe(CN)_6_].

To assess the electrochemical performance of the electrode prepared by our proposed method, a monolithic three-electrode system was laser-patterned on PET. This architecture simultaneously defined the working, counter, and reference electrodes through spatially controlled laser ablation of the gold leaf (Figure 4a). The working electrode had a circular geometric area of approximately 19.6 mm^2^. SEM characterization (Figure S4) revealed a predominantly smooth electrode surface. CV was first performed in 0.5 M H_2_SO_4_ at a scan rate of 0.1 V/s, with the potential ranging from −0.1 V to +1.6 V. As shown in Figure 4b, the CV profile exhibits a prominent oxidation peak with a minor shoulder, followed by a well-defined reduction peak at around +0.58 V corresponding to the reduction of surface gold oxides. Such characteristic gold redox behaviors align closely with those reported for polycrystalline gold electrodes [22]. Next, the operational stability of the gold leaf electrodes was evaluated. Continuous CV cycling (40 cycles) performed in 1 M KCl containing 10 mM K_4_[Fe(CN)_6_] exhibited negligible attenuation in both anodic and cathodic peak currents (Figure 4c), indicating robust stability of the gold leaf electrodes under repeated electrochemical stress. At scan rates ranging from 0.05 to 0.5 V/s, both anodic and cathodic peak currents scale linearly (R^2^ > 0.999) with the square root of scan rates (ν½) (Figure 4d,e). These characteristics indicate diffusion-controlled kinetics, consistent with reported observations on gold leaf electrodes [2,23]. Notably, the gold leaf electrodes maintained stable CV responses after 40 manual bending cycles. CV curves were recorded before bending (0 cycles) and after 10, 20, 30, and 40 bending cycles. The anodic/cathodic peak currents and ΔEp remained nearly unchanged (Figure 4f), indicating mechanical durability. To further validate the reproducibility of the fabrication process, three independently fabricated gold leaf electrodes were tested under identical electrochemical conditions. As shown in Figure 4g, all three electrodes exhibited nearly identical CV profiles, featuring well-defined redox peaks with a peak potential separation (ΔEp) of 77.3 ± 0.58 mV. Additionally, EIS was used to assess electrode reproducibility in 0.1 M KCl containing 10 mM [Fe(CN)_6_]^3−^/^4−^ (Figure 4h). Nyquist plots from three independently fabricated electrodes showed characteristic semicircles and linear tails, reflecting electron-transfer resistance (24.46 ± 0.57 Ω) and diffusion-limited processes, respectively, with nearly superimposable curves indicating excellent inter-electrode consistency.

### 3.3. Voltammetric Characterization of Gold Leaf Electrodes Using UA

**Figure 5:**
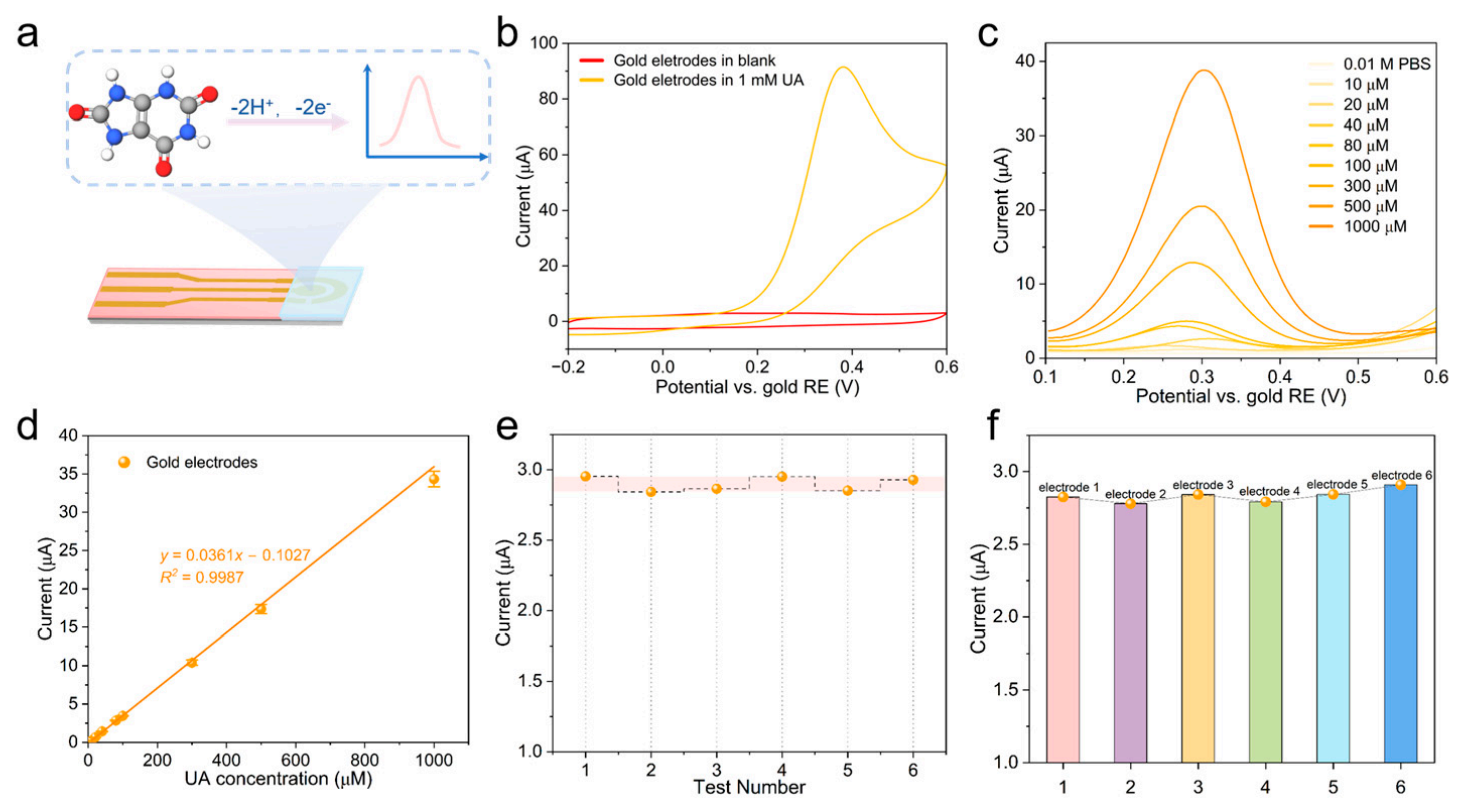
(**a**) Schematic illustration of UA detection on the gold leaf electrodes. (**b**) CV curves of the gold leaf electrodes recorded in blank PBS and in 1 mM UA at 0.1 V/s. (**c**) DPV curves of UA on the gold leaf electrodes. (**d**) Corresponding calibration plot of oxidation peak current vs. UA concentration (*n* = 3). (**e**) Sensor stability assessment via six consecutive DPV runs in 80 μM UA using the same gold leaf electrodes. (**f**) Batch-to-batch reproducibility evaluated by testing six independently fabricated gold leaf electrodes in 80 μM UA.

To further evaluate the electrochemical behavior of our laser-ablated gold leaf electrodes shown in Figure 4a, UA was employed as a model redox-active molecule (Figure 5a). Notably, the unmodified gold leaf electrode lacks the surface functionality and selectivity required for the direct UA analysis of real biological samples. Here, UA was used only to verify the electrochemical responsiveness of the electrodes. As shown in Figure 5b, CV scans in blank PBS solution revealed negligible redox activity. Upon introduction of 1 mM UA, a well-defined oxidation peak appeared at around +0.38 V (vs. gold reference electrode) for the gold leaf electrodes. Then serially diluted UA solutions spanning from 10 μM to 1 mM were analyzed by DPV (Figure 5c,d). The oxidation peak currents of the gold leaf electrodes increased significantly with rising UA concentrations, exhibiting a strong linear correlation (R^2^ > 0.99). Then, intra-electrode repeatability was assessed by six consecutive DPV runs in 80 μM UA using the same electrode (Figure 5e). The response currents exhibited remarkable consistency with a relative standard deviation (RSD) of 1.76%, underscoring good intra-electrode repeatability. Inter-electrode reproducibility was assessed by testing six independently fabricated electrodes in 80 μM UA (Figure 5f). A low RSD of 1.63% in peak currents across electrodes confirmed high batch-to-batch uniformity, demonstrating both reliable fabrication repeatability and outstanding inter-electrode consistency.

### 3.4. Multi-Substrate Fabrication of Gold Leaf Electrodes and Microfluidic Devices

**Figure 6:**
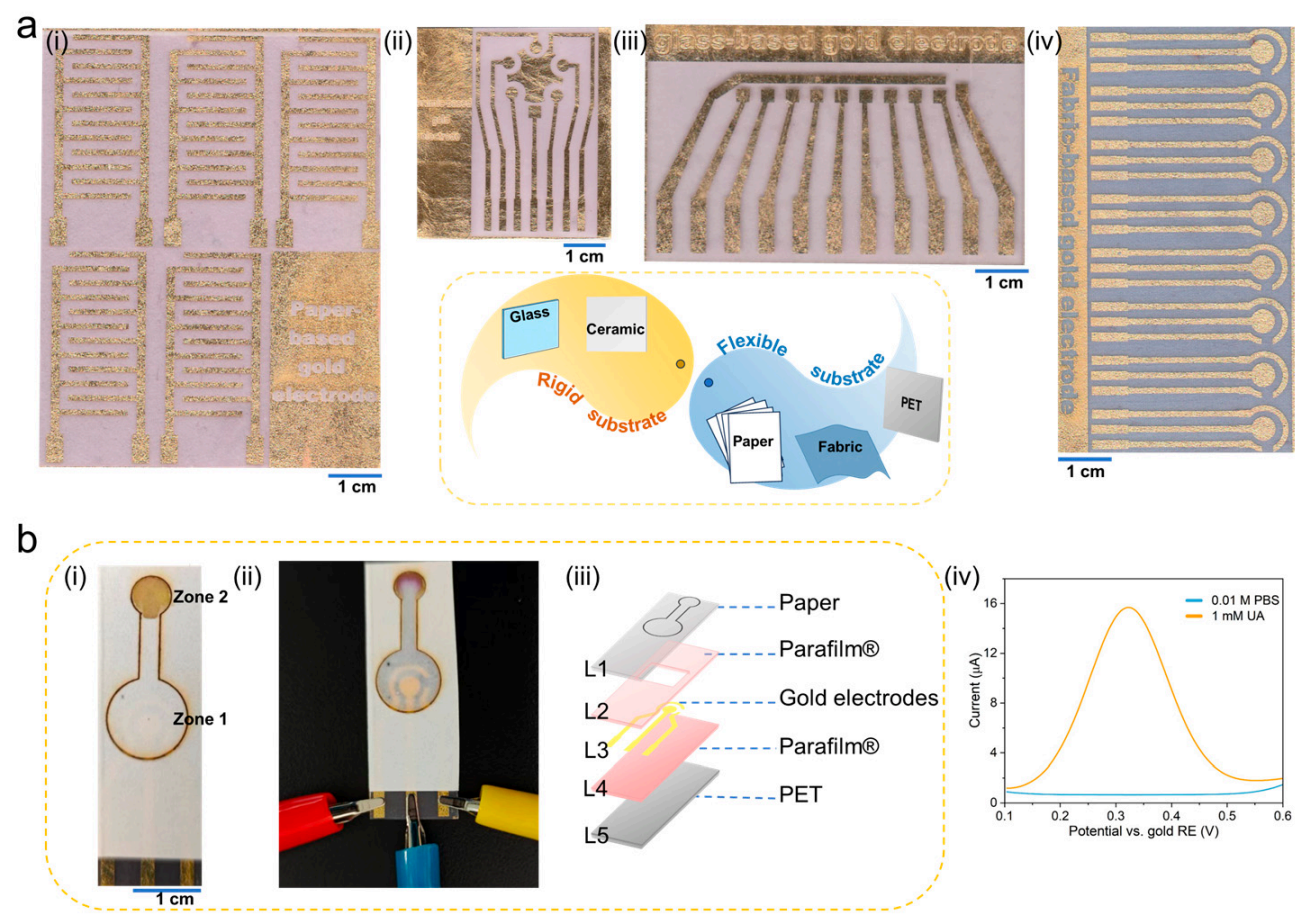
(**a**) Schematic illustration and optical images of patterned gold leaf electrodes on flexible and rigid substrates: (i) paper, (ii) ceramic, (iii) glass, and (iv) fabric. (**b**) Characterization of the paper-based hybrid microfluidic device: optical images of the device (i) before and (ii) after detection of 1 mM UA; (iii) exploded schematic illustrating each layer (L); and (iv) corresponding DPV curves of 1 mM UA.

Building on prior studies demonstrating the versatile adhesive and hydrophobic properties of Parafilm^®^, we further extended the fabrication of gold leaf electrodes to diverse substrates and their integration into microfluidic networks. As shown in Figure 6a, gold leaf was successfully adhered to both flexible (fabric, paper) and rigid (ceramic, glass) substrates through the thermoplastic properties of Parafilm^®^. These substrates enabled precise laser ablation patterning to create functional electrode architectures such as interdigitated electrodes and multi-electrode arrays, achieving multiple electrode fabrication in a single batch. Then, leveraging Parafilm^®^’s capability to form hydrophobic microfluidic barriers, we integrated the fabricated gold leaf electrodes into Parafilm^®^-based microfluidic devices, enabling combined electrochemical sensing and fluidic manipulation. As shown in Figure 6b, an asymmetric dumbbell-shaped microfluidic paper channel was created by Parafilm^®^ thermal bonding and laser ablation, which was then structurally integrated with a laser-patterned gold leaf three-electrode system to assemble the hybrid microfluidic device. The schematics of each layer of the device are provided in Figure S2. The working electrode of the gold-leaf three-electrode system had a circular geometric area of approximately 12.6 mm^2^. The thermal fusion process bonded Parafilm^®^ layer 2 from the paper channel and Parafilm^®^ layer 4 from the gold leaf electrodes, achieving adhesion without secondary adhesives. This configuration established fluidic connectivity between the paper channel and electrodes via plotter-cut Parafilm^®^ windows in layer 2, while defining two spatially separated sensing zones. Upon applying ~25 µL of 1 mM UA to sensing zone 1, downward capillary flow enabled DPV in sensing zone 1, while lateral migration filled sensing zone 2 in approximately 3 min, allowing pH detection via immobilized bromophenol red.

**Figure 7:**
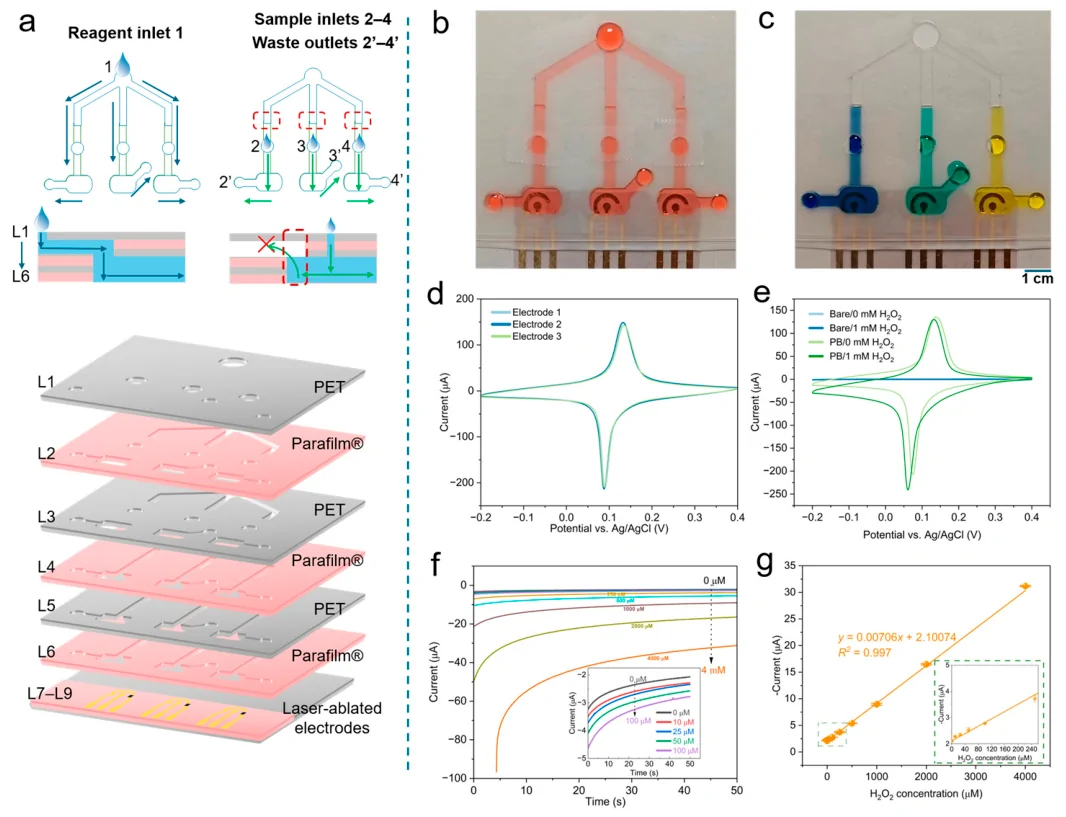
(**a**) Exploded schematic of the PET-based microfluidic device and cross-sectional schematic illustrating sequential flow functions. (**b**,**c**) Optical images after injecting colored food dyes into individual inlets. (**d**) Stabilized cyclic voltammograms of three PB-modified gold leaf working electrodes in 0.1 M KCl + 0.1 M HCl at 50 mV/s. (**e**) Cyclic voltammograms of bare and PB-modified electrodes in 0.1 M KCl + 0.01 M HCl with and without 1 mM H_2_O_2_ at 50 mV/s. (**f**) Amperometric responses of the PB-modified electrode at −0.1 V with varying H_2_O_2_ concentrations. (**g**) Corresponding calibration curve of current response as a function of H_2_O_2_ concentration (*n* = 3). The currents are plotted as their absolute values (i.e., −current) for clarity.

Having validated the hybrid paper-based architecture, we further developed a PET-based microfluidic device by synergistically integrating Parafilm^®^-bonded gold leaf electrodes with structured PET microfluidics (Figure 7a). The schematics of each layer of the device are provided in Figure S3. Through precision plotter cutting of Parafilm^®^ and PET sheets, hierarchical microchannel architectures were created through a simple heat-pressing step. Sequential flow control was realized not only by tuning the number of Parafilm^®^ and PET layers, but also by the three-dimensional stacking arrangement of the pre-patterned channels across these layers. As demonstrated with food dye solutions, fast reagent loading was achieved in approximately 10 s using a homemade adapter that connected the Pasteur pipette to the inlet (Figure 7b and Figure S5). During sample loading, the normally tape-sealed sample inlet was uncovered and used for injection; the flow toward the reagent inlet was impeded due to the upward path and the hydrophobic Parafilm^®^ floor it would have to overcome. The colored solution preferentially entered the electrochemical cell capable of holding ~250 μL of liquid (Figure 7c).

While PB-modified electrodes have demonstrated wide applicability in H_2_O_2_ sensing across numerous substrates (e.g., glassy carbon, pyrolytic carbon, carbon paste, sputtered gold, nanoporous gold) [24,25,26,27,28], to the best of our knowledge, the electrochemical behavior of PB deposited on gold leaf has not been reported. Thus, we employed PB electrodeposition, electrode characterization, and H_2_O_2_ detection as a model workflow to demonstrate the device’s capability for sequential reagent delivery and on-chip electrochemical processing. In this device, the gold reference electrode was converted to an Ag/AgCl reference electrode by coating with commercial Ag/AgCl paste, thereby ensuring a stable reference potential for PB electrodeposition and constant-potential amperometry. Leveraging the common reagent inlet, sequential PB deposition and electrochemical activation in a single run delivered three PB-modified gold leaf working electrodes. As shown in Figures S6 and S7, SEM imaging revealed a nanoparticle-based film on the modified electrode surface, with some nanoparticles forming clusters. EDS elemental mapping detected Fe, C, N, and K distributed across the surface, which is consistent with the elemental composition expected for a PB deposit. Together with the characteristic reversible PB/Prussian white redox peaks (Figure S8) and the electrocatalytic response toward H_2_O_2_ at −0.1 V on the electrode (Figure 7e), these results support the successful electrodeposition of PB on the gold leaf electrode. Furthermore, highly overlapping voltammograms across the three PB-modified electrodes demonstrated excellent interelectrode reproducibility of the electrodeposition process within the microchannel-based cells (Figure 7d). Following characterization, the PB-modified electrodes were applied to H_2_O_2_ detection. Considering that PB is hydrolytically unstable above approximately pH 7 [29], the acidified supporting electrolytes were therefore deliberately selected to keep PB stable during sensing. By selective sample loading through dedicated inlet/outlet pairs, the device allowed 1–3 replicate measurements per sample or parallel analysis of up to three distinct samples, without cross-filling unused electrochemical cells. As shown in Figure 7e, CV curves of the bare gold leaf electrode in 0 and 1 mM H_2_O_2_ remain largely unchanged. By contrast, the PB-modified gold leaf electrode exhibits a markedly increased cathodic current in the presence of 1 mM H_2_O_2_, indicating a PB-mediated catalytic effect. Further amperometric measurements of H_2_O_2_ standards at −0.1 V showed a concentration-dependent increase in cathodic current, affording a linear calibration curve with a limit of detection (LOD) of 12.98 µM (equivalent to 0.44 mg/L) (Figure 7f,g). The LOD was calculated as 3σ/S, where S is the slope of the calibration curve and σ is the standard deviation of the current response of the blank solution. It falls within a reasonable range for PB-based H_2_O_2_ sensors on planar electrodes (Table S3) [30,31,32,33,34,35]. The amperometric response remained virtually unchanged in the presence of glucose or dopamine at equal concentration, or L-ascorbic acid at half concentration of H_2_O_2_ (Figure S9a). In addition, common water ions such as Na^+^, Mg^2+^, Ca^2+^, and NO_3_^−^ at 10-fold concentration relative to H_2_O_2_ caused no obvious interference (Figure S9b). These results indicate that the PB-modified gold leaf electrode has acceptable interference tolerance for the tested common species. In real environmental samples, we performed recovery experiments using pond water spiked with known concentrations of H_2_O_2_. For all three spiked levels, recoveries were approximately 85% with RSDs < 4.5% (Figure S10). This moderate recovery likely reflects matrix effects and systematic losses. Sample pretreatment or selective coatings on the electrode could further improve accuracy in future applications.

## 4. Conclusions

In summary, while gold-leaf electrodes and Parafilm^®^-based microfluidics have been reported separately, this study demonstrates their integration into a single Parafilm^®^-mediated fabrication and microfluidic platform. Parafilm^®^ simultaneously acts as a thermoplastic adhesive for gold-leaf patterning and as a channel-defining/bonding layer, enabling cross-substrate microfluidic–electrochemical integration without additional interfacial adhesives. By combining Parafilm^®^’s thermoplastic adhesion with laser ablation, we fabricated patterned gold leaf electrodes on flexible PET substrates, exhibiting good mechanical durability and stable electrochemical performance. The approach’s versatility spans both material diversity and substrate generality, with laser ablation enabling precise patterning across these configurations. Building on this, Parafilm^®^ as an adhesive interlayer and microfluidic barrier enabled cross-domain integration of electrodes and fluidic networks. By eliminating the need for secondary adhesives or complex surface treatments, the Parafilm^®^-mediated platform can accelerate prototyping and reduce fabrication costs for microfluidic–electrode hybrids on both flexible and rigid substrates. Successful demonstrations on both paper–PET hybrid and PET-based microfluidic devices with embedded electrodes underscore the versatility of our strategy for a range of multifunctional microfluidic sensing systems.

## Data Availability

The original contributions presented in this study are included in the article. Further inquiries can be directed to the corresponding author.

## Supplementary Materials

The following supporting information can be downloaded at: https://www.mdpi.com/article/10.3390/mi17101136/s1. Figure S1. Cyclic voltammograms of the gold leaf electrodes recorded in 10 mM K_4_[Fe(CN)_6_]/1 M KCl and in PBS. Figure S2. Layer-by-layer planar views and dimensional schematic of the paper-based hybrid device. Figure S3. Layer-by-layer planar views and dimensional schematic of the PET-based microfluidic device. Figure S4. SEM image of the gold leaf electrode. Figure S5. Optical image showing the loading of a food dye solution into the microfluidic device. Figure S6. SEM image of the PB-modified working electrode. Figure S7. SEM image and corresponding energy-dispersive X-ray spectroscopy elemental maps of the PB-modified working electrode. Figure S8. Overlaid cyclic voltammograms of the PB-modified electrode in 0.1 M KCl + 0.1 M HCl at 0.05 V/s. Figure S9. Amperometric responses of the PB-modified electrode to 200 µM H_2_O_2_ alone and in the presence of various interfering species. Figure S10. Recoveries of H_2_O_2_ in spiked pond water samples. Table S1. Summary of the designed and achieved line width of gold leaf line patterns. Table S2. Summary of the designed and achieved pitch on gold leaf. Table S3. Comparison of H_2_O_2_ sensors based on gold electrodes or on planar electrodes modified with PB.
