# Supplementary material for: Universal Parafilm®-Mediated Thermal Bonding for Gold Leaf Electrode Patterning and Microfluidic Assembly on Diverse Substrates
Source: Micromachines (Basel). 2026 Sep 29;17(10):1136. doi: 10.3390/mi17101136 (PMC13707072; doi:10.3390/mi17101136)
Supplement: Supplementary file 1 [file micromachines-17-01136-s001.zip › micromachines-4538015-supplementary.pdf]

Supplementary materials

Universal Parafilm®-Mediated Thermal Bonding for Gold Leaf Electrode Patterning and Microfluidic Assembly on Diverse Substrates

Zhiyuan Yang[1], Yafei Lou[2], Siyu Chen[2], Yongye Chen[2], Linan Sun[2], Junfei Tian[2], Rong Cao[1,*]

[1] NHC Key Laboratory of Tropical Disease Control, School of Life Sciences and Medical Technology, Hainan Medical University, Haikou, Hainan, 571199, China

[2] State Key Laboratory of Pulp and Paper Engineering, School of Light Industry and Engineering, South China University of Technology, Guangzhou 510640, China

*Corresponding author.

E-mail addresses: caorong_chem@163.com (R. Cao).

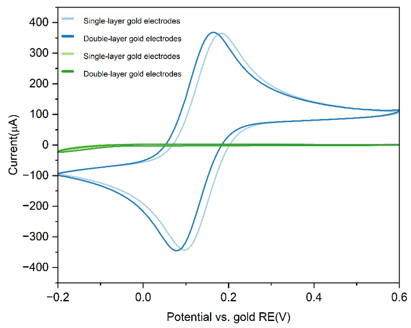

Figure S1. Cyclic voltammograms of the gold leaf electrodes recorded in 10 mM $K_4[Fe(CN)_6]$/1 M KCl (blue) and in PBS (green).

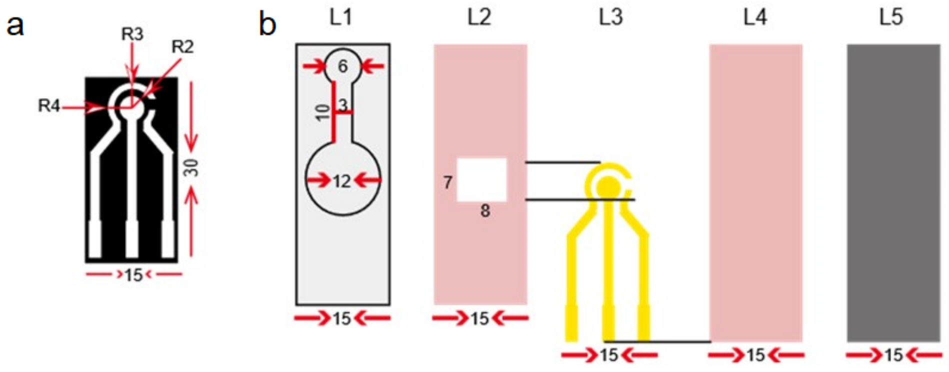

Figure S2. Layer-by-layer planar views and dimensional schematic of the paper-based hybrid device. (a) Gold leaf electrode dimensions. (b) Planar layouts of layers (L1–L5). L1 is the laser-ablated patterned paper layer; L2 is the Parafilm® layer with a plotter-cut pattern; L3 is the gold leaf electrode layer; L4 is the Parafilm® layer; L5 is the PET supporting layer. (Unit: mm).

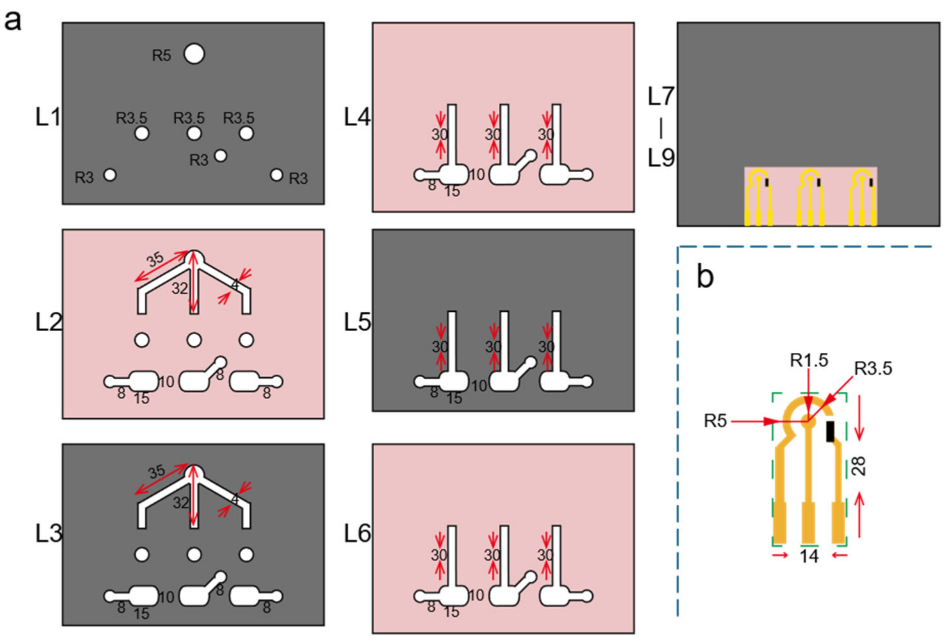

Figure S3. Layer-by-layer planar views and dimensional schematic of the PET-based microfluidic device. (a) Planar layouts of layers (L1–L9). L1 is the top PET layer patterned with a reagent inlet, three sample inlets, and three waste outlets. L2 is the Parafilm® layer with plotter-cut patterns; L3 is the PET layer with plotter-cut patterns; L4 is the Parafilm® layer with plotter-cut patterns; L5 is the PET layer with plotter-cut patterns; L6 is the Parafilm® layer with plotter-cut patterns; L7–L9 constitute a laser-ablated electrode assembly. (b) Gold leaf electrode dimensions. The Ag/AgCl reference electrode is indicated. (Unit: mm).

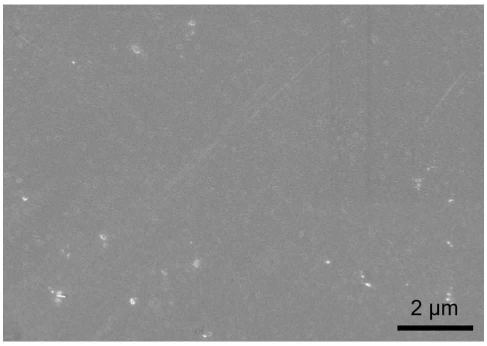

Figure S4. SEM image of the gold leaf electrode.

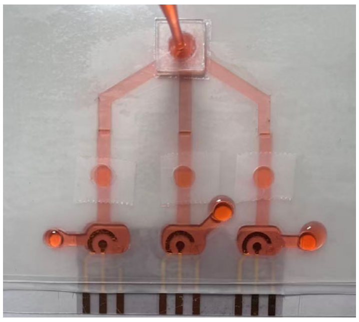

Figure S5. Optical image showing the loading of a food dye solution into the microfluidic device. A plastic Pasteur pipette was connected to the reagent inlet *via* a homemade adapter, allowing the dye solution to fill the entire channel network.

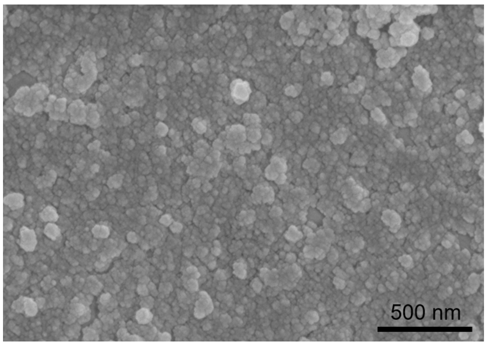

Figure S6. SEM image of the PB-modified working electrode.

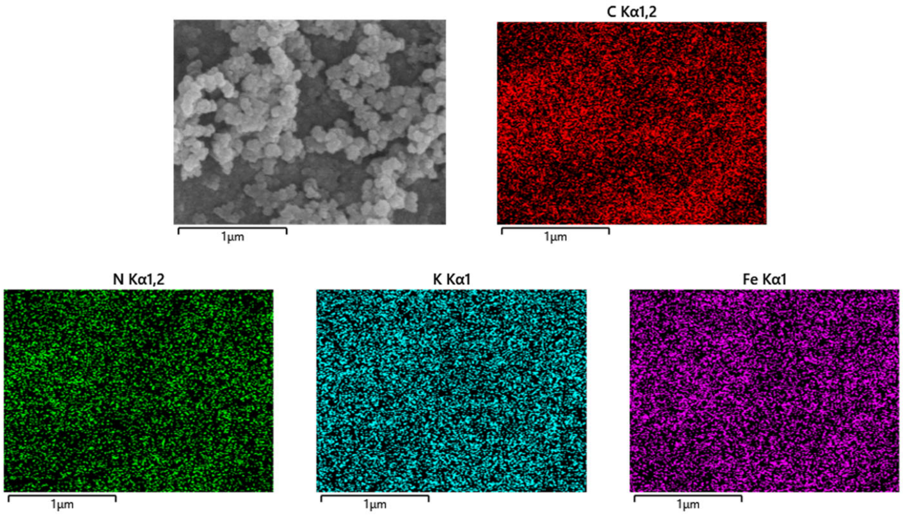

Figure S7. SEM image and corresponding energy-dispersive X-ray spectroscopy elemental maps (C, N, K, Fe) of the PB-modified working electrode.

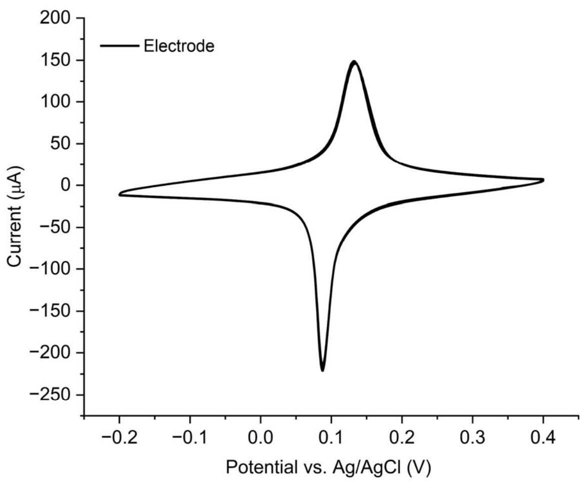

Figure S8. Overlaid cyclic voltammograms of the PB-modified electrode in 0.1 M KCl + 0.1 M HCl at 0.05 V/s.

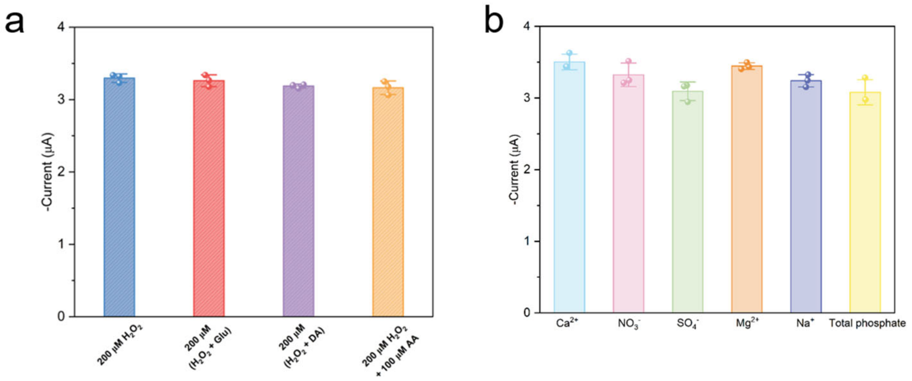

Figure S9. (a) Amperometric responses of the PB-modified electrode to 200 μM H_2O_2 alone and in the presence of 200 μM glucose (Glu), 200 μM dopamine (DA), or 100 μM L-ascorbic acid (AA) (*n* =3). **(b)** Amperometric responses of the PB-modified electrode to 200 μM H_2O_2 in the presence of 2 mM various interfering species (Ca^{2+}, NO_3^-, SO_4^{2-}, Mg^{2+}, Na^+ and total phosphate), corresponding to a 10-fold excess over H_2O_2 (*n* =3). The reduction currents are plotted as their absolute values (*i.e.*, −current) for clarity.

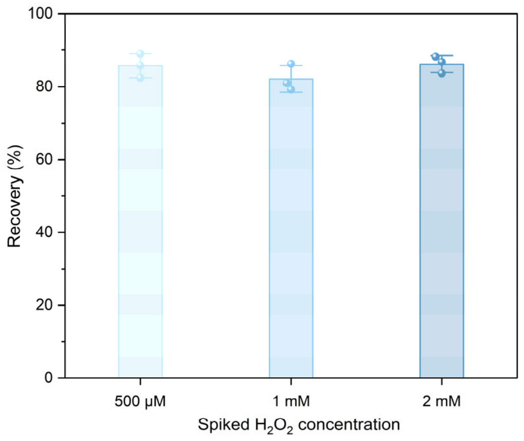

Figure S10. Recoveries of H_2O_2 in spiked pond water samples (n =3).

Table S1 Summary of the designed and achieved line width (LW) of gold leaf line patterns (n = 3).

Designed LW (mm)	Achieved LW (mm)
0.1	0.053 ± 0.003
0.2	0.130 ± 0.006
0.3	0.264 ± 0.007
0.4	0.346 ± 0.002
0.5	0.467 ± 0.006
0.6	0.542 ± 0.004
0.7	0.668 ± 0.007
0.8	0.750 ± 0.003
1.0	0.953 ± 0.010
1.2	1.156 ± 0.007

Table S2 Summary of the designed and achieved pitch on gold leaf (n = 3).

Design pitch (mm)	Achieved pitch (mm)
0.05	0.069 ± 0.006
0.1	0.118 ± 0.004
0.2	0.213 ± 0.006
0.3	0.301 ± 0.005
0.4	0.408 ± 0.005
0.5	0.496 ± 0.005
0.6	0.607 ± 0.007
0.7	0.704 ± 0.003
0.8	0.798 ± 0.002

Table S3 Comparison of H_2O_2 sensors based on gold electrodes or on plana electrodes modified with PB.

Analyte	Working electrode	Detection mode	Detection buffer	Detection potential	Limit of detection	Ref.
Hydrogen peroxide	Nanoporous gold film electrode on a gold substrate	Amperometry	PBS, pH 7	−0.2 V	3.7 μM	1
Hydrogen peroxide	Bare gold leaf electrode	Amperometry	PBS, pH 7.4	−0.45 V	0.713 mM	2
Hydrogen peroxide	Laser-induced graphene electrode coated with chitosan and PB	Amperometry	Britton–Robinson buffer, pH 6	−0.036 V	6.31 μM	3
Hydrogen peroxide	Prussian blue bulk-modified carbon screen-printed electrode	Amperometry	PBS, pH 7.4	0 V	0.4 μM	4
Hydrogen peroxide	Carbon screen-printed electrode modified with inkjet-printed PB	Amperometry	0.05 M phosphate buffer containing 0.1 M KCl, pH 7.4	0 V	0.2 μM	5
Hydrogen peroxide	Laser-induced graphene coated with chemically deposited PB and chitosan	Amperometry	PBS, pH 7.4	−0.1 to 0.1 V	121 μM	6
Hydrogen peroxide	PB-modified gold leaf electrode	Amperometry	0.1 M KCl + 0.01 M HCl	−0.1 V	12.98 μM	This work

References

[1] Sukeri, A.; Lima, A. S.; Bertotti, M. Development of non-enzymatic and highly selective hydrogen peroxide sensor based on nanoporous gold prepared by a simple unusual electrochemical approach. Microchem. J. 2017, 133, 149−154.

[2] Mohd Asri, M. A.; Mak, W. C.; Azizah, S.; Nordin, A. N. Low-cost and rapid prototyping of integrated electrochemical microfluidic platforms using consumer-grade off-the-shelf tools and materials. Lab Chip 2022, 22, 1779−1792.

[3] Barber, R.; Davis, J.; Papakonstantinou, P. Stable chitosan and Prussian Blue-coated laser-induced graphene skin sensor for the electrochemical detection of hydrogen peroxide in sweat. ACS Appl. Nano Mater. 2023, 6, 10290−10302.

[4] O'Halloran, M. P.; Pravda, M.; Guilbault, G. G. Prussian Blue bulk modified screen-printed electrodes for H_2O_2 detection and for biosensors. Talanta 2001, 55, 605−611.

[5] Cinti, S.; Arduini, F.; Moscone, D.; Palleschi, G.; Killard, A. J. Development of a hydrogen peroxide sensor based on screen-printed electrodes modified with inkjet-printed Prussian Blue nanoparticles. Sensors 2014, 14, 14222−14234.

[6] Bauer, M.; Wunderlich, L.; Weinzierl, F.; Lei, Y.; Duerkop, A.; Alshareef, H. N.; Baeumner, A. J. Electrochemical multi-analyte point-of-care perspiration sensors using on-chip three-dimensional graphene electrodes. Anal. Bioanal. Chem. 2021, 413, 763−777.
